# Apigenin Attenuates Acetaminophen-Induced Hepatotoxicity by Activating AMP-Activated Protein Kinase/Carnitine Palmitoyltransferase I Pathway

**DOI:** 10.3389/fphar.2020.549057

**Published:** 2020-11-20

**Authors:** Jiaqi Zhang, Xiaoqiang Liang, Jiacheng Li, Hao Yin, Fangchen Liu, Cheng Hu, Ling Li

**Affiliations:** ^1^ Shanghai TCM-Integrated Institute of Vascular Anomalies, Shanghai TCM-Integrated Hospital, Shanghai University of Traditional Chinese Medicine, Shanghai, China; ^2^ Longhua Hospital Affiliated to Shanghai University of Traditional Chinese Medicine, Shanghai, China; ^3^ Department of Neurology, Shanghai TCM-Integrated Hospital, Shanghai University of Traditional Chinese Medicine, Shanghai, China; ^4^ Experiment Center for Science and Technology, Shanghai University of Traditional Chinese Medicine, Shanghai, China

**Keywords:** apigenin, metabolomics, carnitine palmitoyltransferase I, AMP-activated protein kinase, acetaminophen-induced hepatotoxicity

## Abstract

Overuse of acetaminophen (APAP) is a major cause of drug-induced liver failure at the clinics. Apigenin (API) is a natural flavonoid derived from *Matricaria chamomilla.* The aim of the present study was to investigate the amelioration function of API in APAP-induced hepatotoxicity both *in vitro* and *in vivo* and investigate its potential mechanisms. Analysis results of the activities of serum alanine and aspartate aminotransferases (ALT and AST), malondialdehyde, myeloperoxidase (MPO), and reactive oxygen species (ROS) demonstrated therapeutic effects of API. MTT assay results revealed that API attenuated APAP and its metabolic product, N-acetyl-p-benzoquinone imine (NAPQI) induced cytotoxicity in a dose-dependent manner in human liver cells, L-02 cells. Subsequently, metabolomic results of cells and serum analyses demonstrated an aberrant level of carnitine palmitoyltransferase I (CPT1A). We established that API stimulated CPT1A activity in mice liver tissues and L-02 cells. Molecular docking analyses revealed potential interaction of API with CPT1A. Further investigation of the role of CPT1A in L0-2 cells revealed that API reversed cytotoxicity *via* the AMP-activated protein kinase (AMPK)/GSK-3β signaling pathway and compound C, which is a selective AMPK inhibitor, inhibited activation of CPT1A induced by API. API was bound to the catalytic region of AMPK as indicated by molecular docking results. In addition, compound C suppressed nuclear translocation of nuclear factor erythroid 2–related factor 2 (NRF2) that is enhanced by API and inhibited the antioxidative function of API. In summary, the study demonstrates that API attenuates APAP-induced hepatotoxicity by activating the AMPK/GSK-3β signaling pathway, which subsequently promotes CPT1A activity and activates the NRF2 antioxidant pathway.

## Introduction

Acetaminophen (APAP, paracetamol) is a widely used over-the-counter analgesic and antipyretic drug. While APAP has a few toxic side effects at normal doses, prolonged or excessive use of APAP can lead to inflammatory infiltration, degeneration, and necrosis of hepatic lobules in animals and humans (ADDIN EN.CITE). An overdose of APAP is the primary cause (over 60%) of drug-induced liver injury (DILI), which is a leading cause of acute liver failure (ALF) in developed countries (Nourjah et al., 2006).

APAP forms a highly reactive metabolite, N-acetyl-p-benzoquinone imine (NAPQI) *via* the activity of CYP450 enzyme (mainly CYP2E1 in human body), which induces hepatotoxicity (Huggett and Blair, 1983). Although cellular reduced glutathione (GSH) can detoxify the hepatotoxicity (Mitchell et al., 1973; Kanno et al., 2017), once an overdose of APAP is ingested and excess NAPQI formed, GSH is depleted (van de Straat et al., 1987; Duan et al., 2019). The superabundant NAPQI then covalently binds to the protein sulfur group to form protein conjugates (Jollow et al., 1973), which could cause intracellular mitochondrial and DNA damage and oxidative stress, eventually leading to liver necrosis. Research on finding the cure for liver damage caused by APAP overdose has increased in the recent past. N-acetylcysteine is used to treat APAP-induced hepatic injury (Corcoran and Wong, 1986). The primary function of the drug is to maintain and supplement GSH content in the liver, enhance detoxification ability of the body to NAPQI, and prevent damage to normal liver cells (Wang et al., 2006; Green et al., 2013). However, previous studies have revealed that N-acetylcysteine has numerous disadvantages, such as poor or delayed treatment and gastrointestinal adverse reactions, among others (Kerr et al., 2005; Whyte et al., 2010). Therefore, it is imperative to develop new, safe and protective treatment agents for APAP-induced hepatotoxicity.

Apigenin (API) or versulin (4′,5,7-trihydroxyflavone) is a natural flavonoid derived from *Matricaria chamomilla* with high antioxidant capacity. Previous studies have revealed that flavonoids can protect against different types of hepatopathy through anti-inflammatory and antioxidant mechanisms, especially hepatopathy that is associated with AP-induced liver damage (Jing et al., 2018; Shi et al., 2018; Zhao et al., 2019). In addition, previous studies have demonstrated that API can protect against various liver injuries caused by alcohol (Wang et al., 2017), lipopolysaccharides (Zhou et al., 2017), and ischemia/reperfusion (Tsaroucha et al., 2016). In the present study, we investigated the protective mechanisms of API against APAP-induced liver injury.

## Results

### Apigenin Enhanced Acetaminophen-Induced Hepatotoxicity Both *In Vivo* and *In Vitro*

**FIGURE 1:**
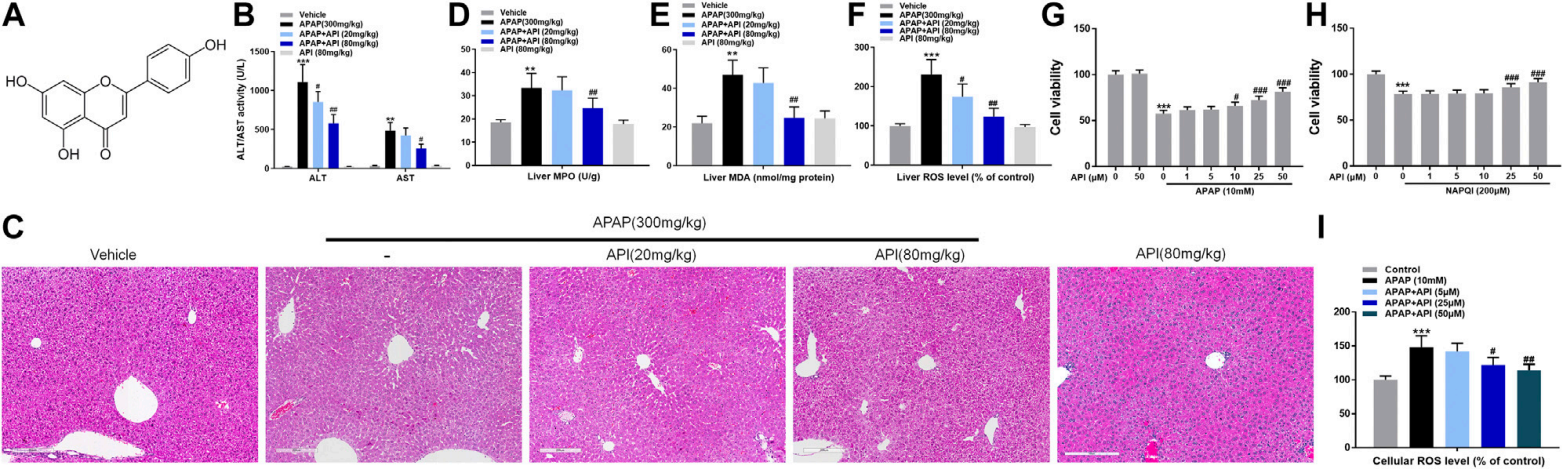
Apigenin (API) ameliorated acetaminophen (APAP)-induced hepatotoxicity in C57BL/6J mice and attenuated APAP-induced cytotoxicity in L-02 cells. **(A)** Structure of API. The molecular formula of API is C_15_H_10_O_5_ (molecular weight = 270.24). **(B)** Alanine aminotransferase and aspartate aminotransferase activities. **(C)** Representative images of H&E-stained liver sections (magnification of ×200). **(D)** Liver myeloperoxidase levels. **(E)** Liver malondialdehyde activity. **(F)** Liver reactive oxygen species (ROS) levels. **(G,H)** Cell viability evaluated by MTT assay in response to APAP (10 mM) or N-acetyl-p-benzoquinone imine (200 μM) treatment of API in L-02 cells at different time intervals. **(I)** Cell ROS levels. Data are expressed as means ± SEM (n = 6 in mice and n = 3 in L-02 cells); **p < 0.01, ***p < 0.001 compared to the control group; #*p* < 0.05, ##*p* < 0.01, ###*p* < 0.001 compared to the APAP group).

To investigate the therapeutic detoxification capacity of API against APAP-induced hepatotoxicity, an animal model of APAP-induced liver injury was constructed in which C57BL/6J mice were orally given APAP at a dose of 300 mg/kg. API (20 or 80 mg/kg) was subsequently administered orally 4 h after treatment with APAP, and liver tissues and serum were collected 4 h after treatment with API. Afterward, a series of cellular damage and oxidative stress injury parameters were examined. APAP increased alanine aminotransferase and aspartate aminotransferase activities, liver malondialdehyde (MDA), myeloperoxidase (MPO), and reactive oxygen species (ROS) levels, whereas treatment with API reversed APAP-induced hepatotoxicity *in vivo* (Figures 1B–F). H&E staining results revealed that intrahepatic hemorrhage and destruction of liver structures occurred in the APAP group, but the symptoms were ameliorated by API (Figure 1C). Besides, we examined the concentration of immune activation markers interleukin-6 (IL-6) and tumor necrosis factor alpha (TNF-α) in serum and mRNA levels in mice liver tissues. The results showed APAP increased the level of IL-6 and TNF-α both in serum and liver tissues and API reversed it (Supplementary Figures S1A,B). To explore the time course of the damage caused by APAP, we collected mice liver tissues and serum 0–8 h after treatment with API. Supplementary Figures S1C–E results revealed that in 0 h after API administration, API has not had enough time to exert therapeutic effect. It is noteworthy that API ameliorated APAP-induced liver injury after 8 h administration (Supplementary Figures S1F–H).

Subsequently, a cellular APAP hepatocyte toxicity model was constructed by incubating different concentrations of API in normal human liver cells, L-02, for 15 min, followed by incubation of APAP or its metabolite, NAPQI, for 24 h. Cell viability of L-02 cells was evaluated using MTT assay, and the results revealed that API not only enhanced hepatotoxicity caused by APAP and its metabolites but also enhanced hepatotoxicity caused solely by NAPQI, both in a dose-dependent manner (Figures 1G,H). ROS results (Figure 1I) revealed that oxidative stress injury was enhanced. Overall, the results demonstrated that API enhances APAP-induced hepatotoxicity both *in vivo* and *in vitro.*

### Identification of Orthogonal Partial Least Squares-discrimination analysis (OPLS-DA) and Metabolites

**FIGURE 2:**
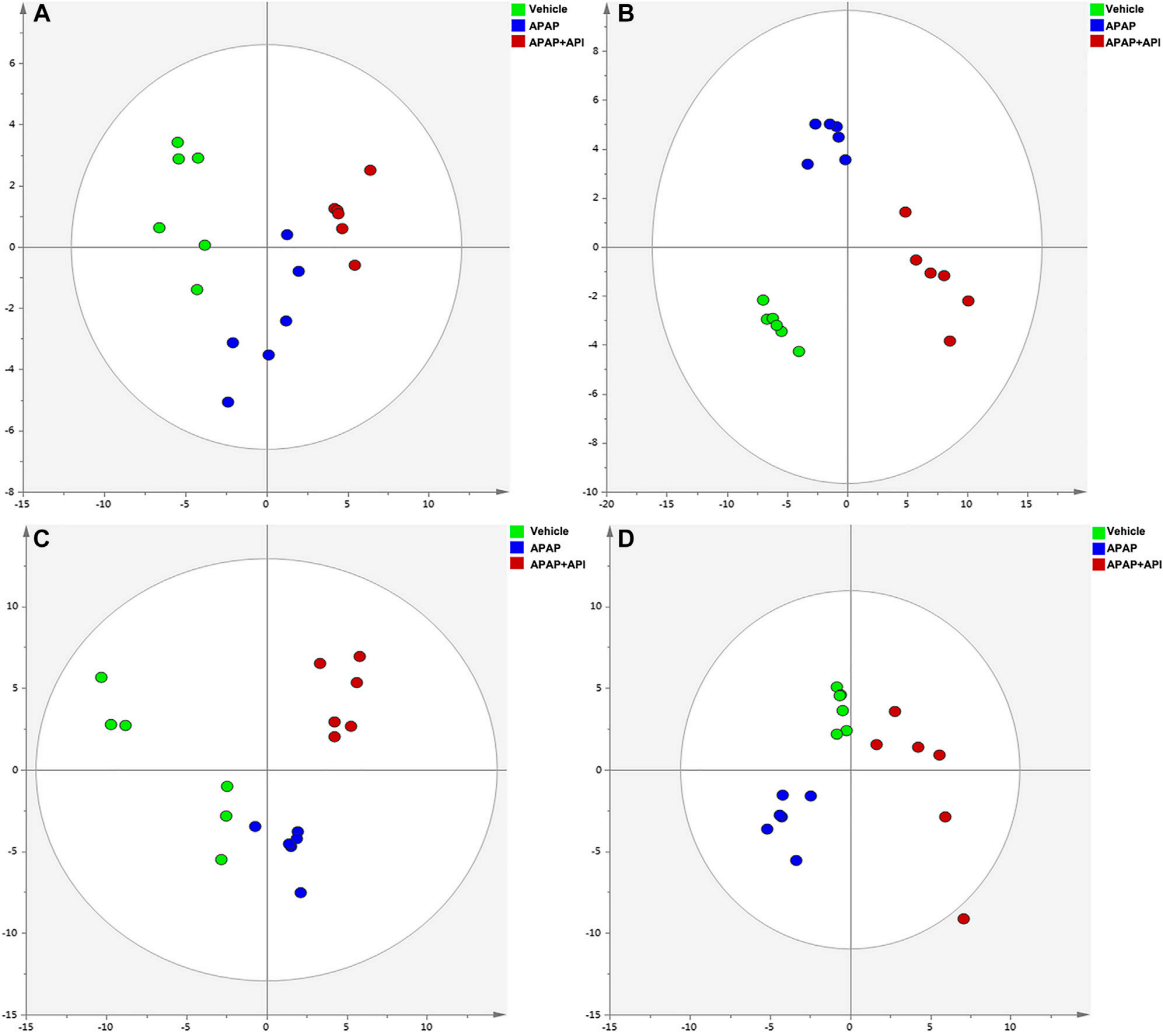
PCA of cell and serum metabolites (n = 6). **(A)** PCA score plot of L-02 cells in negative mode. **(B)** PCA score plot of L-02 cells in positive mode. **(C)** PCA score plot of L-02 serum in negative mode. **(D)** PCA score plot of serum in positive mode.

The R^2^X and Q^2^Y values for Principal Component Analysis (PCA) performed on cell and serum samples in positive and negative modes were over 0.741 and 0.781, respectively (Figure 2), which indicated that our analytical model was of reliable discriminative and predictive degree.

**FIGURE 3:**
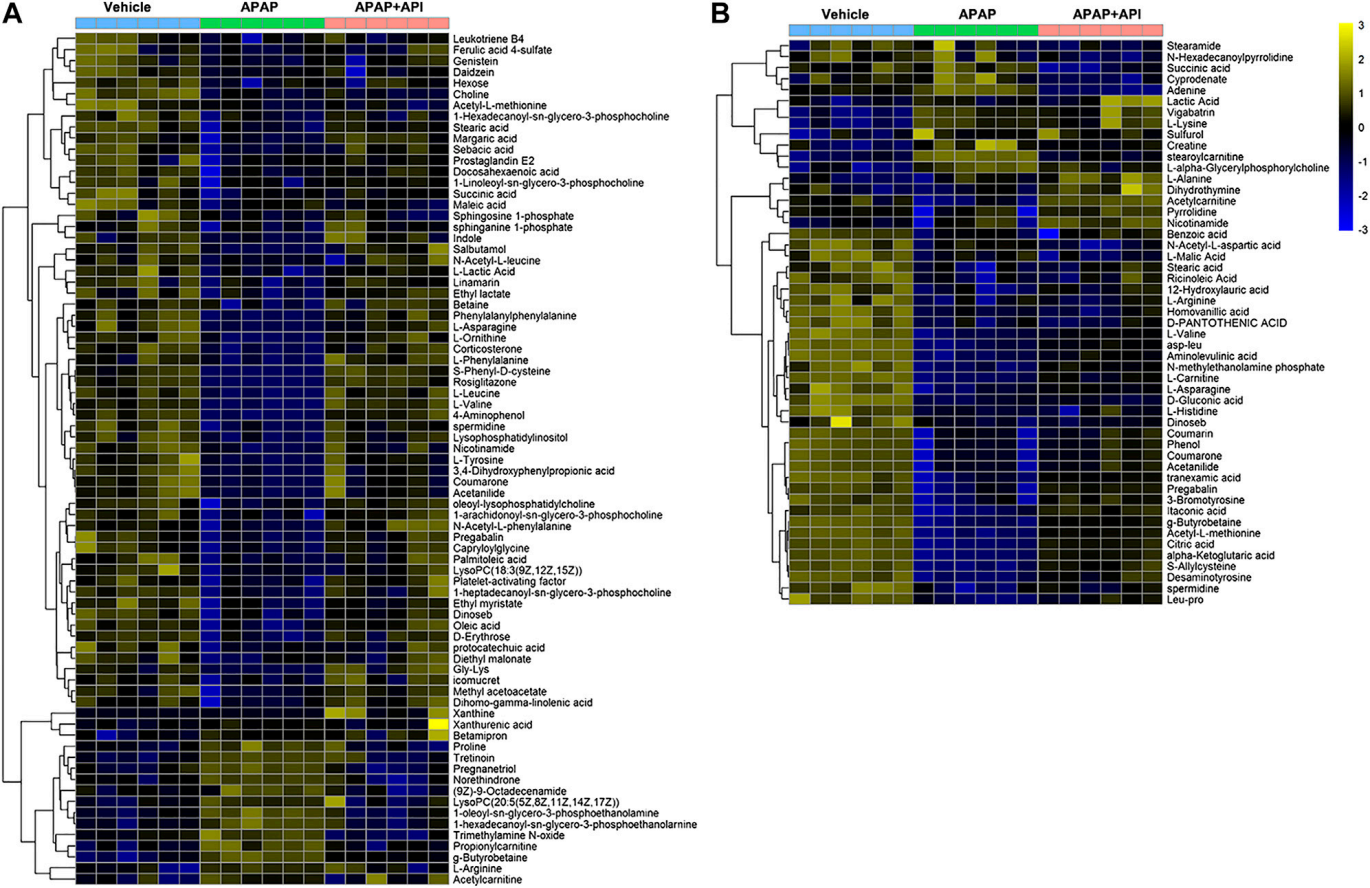
Heat map of identified metabolites in L-02 cells and serum samples. **(A)** Serum of C57BL/6J mice. **(B)** L-02 cells. **(C)** Related signaling pathway.

OPLS-DA was performed using APAP and control groups to determine potential differential metabolites, and variable importance in projection (VIP >1) and *p*-value (*p* < 0.01) was used for assessment. Finally, 77 different metabolites in serum samples and 51 metabolites in cell samples were identified by searching MS libraries. The treatment trend of API is presented in Figures 3A,B. The heat map reveals that almost all metabolites in mice serum and nearly two-thirds of the metabolites in cells returned to normal levels.

**FIGURE 4:**
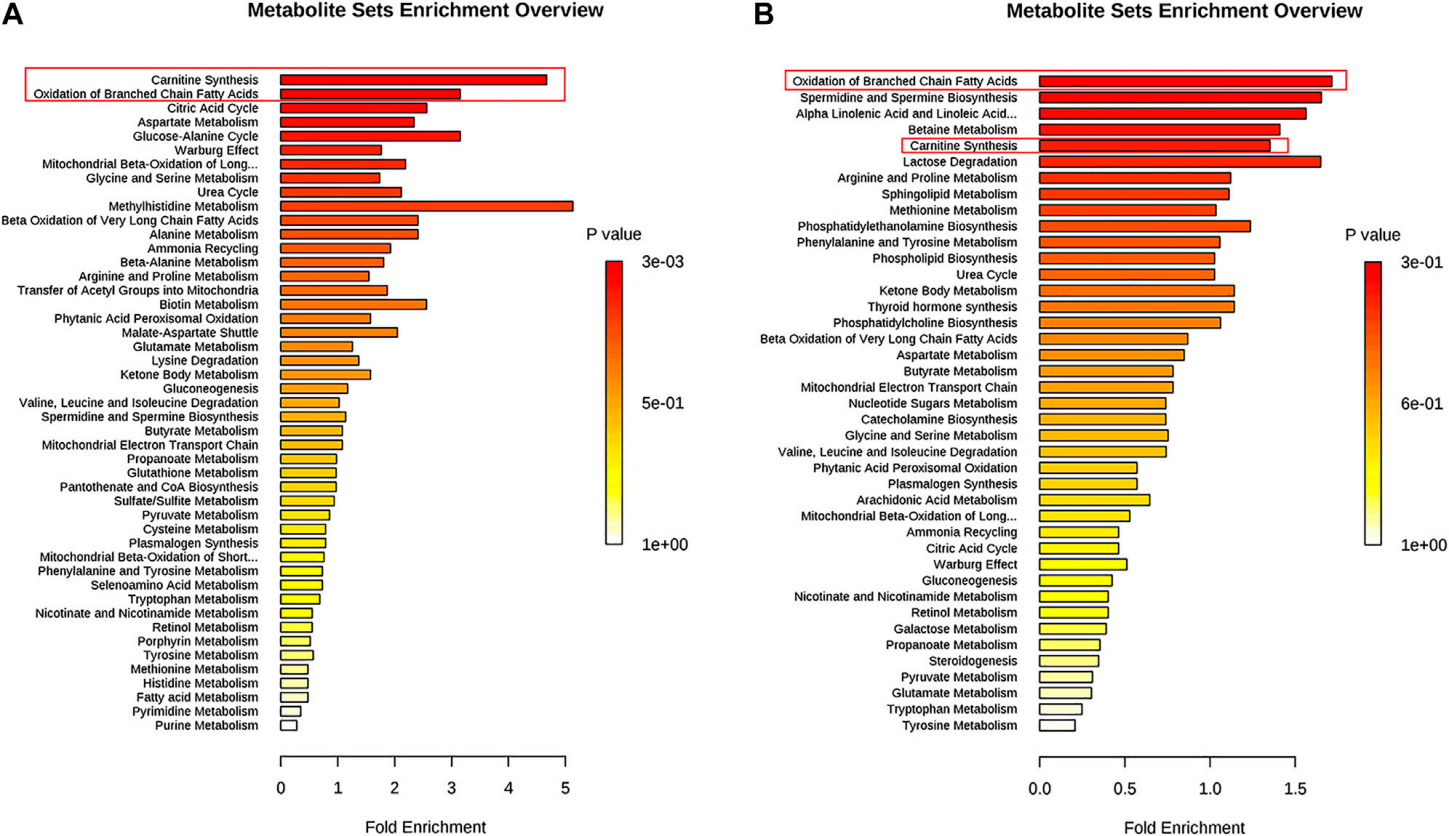
Pathway enrichment of significantly altered metabolites in L-02 cells and serum. **(A)** L-02 cells. **(B)** Serum of C57BL/6J mice.

Metabolites (VIP >1, *p* < 0.01) were analyzed by MetaboAnalyst to enrich potential pathways. Results of the analyses revealed that carnitine synthesis and oxidation of fatty acids played crucial roles in APAP-induced liver injury (Figure 4). Based on the KEGG and SMPDB databases, we established that a change in the metabolite pathway was associated with the components of the CPT1 signaling pathway (Figure 4).

### Apigenin Enhanced Carnitine Palmitoyltransferase I Activity Both *In Vitro* and *In Vivo*

Certain relative parameters of carnitine palmitoyltransferase I (CPT1A) were examined to verify the relationship between API and CPT1A. Results of the analyses revealed that API enhanced CPT1A activity, mRNA expression of CPT1A, and protein expression of CPT1A in liver tissues, although treatment with APAP alone had no effect (Figures 3A–C), which demonstrated that API enhanced CPT1A activity *in vivo*.

**FIGURE 5:**
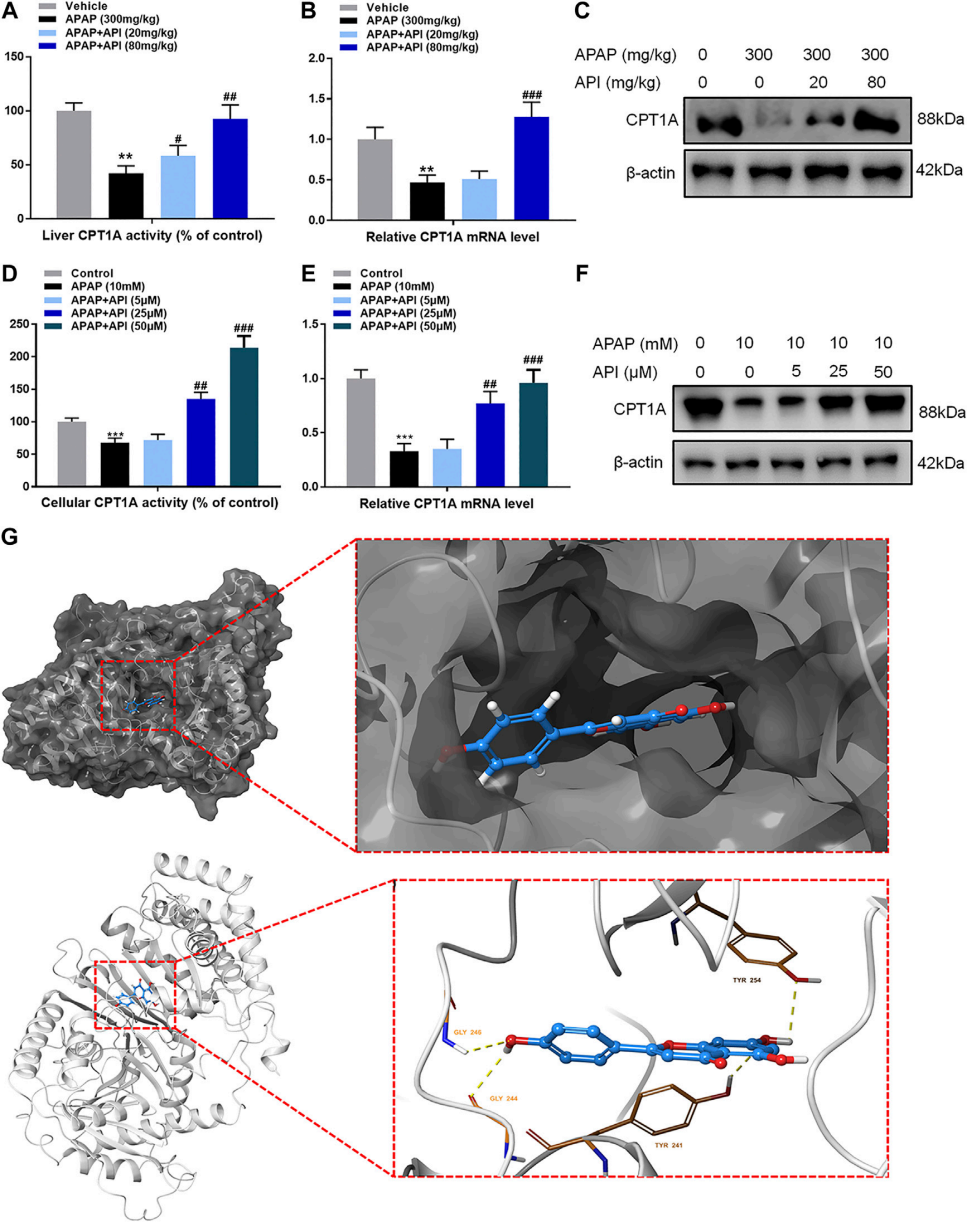
Apigenin (API) enhanced carnitine palmitoyltransferase I (CPT1A) activity both *in vitro* and *in vivo*. **(A)** CPT1A activity in the liver. **(B)** mRNA expression of CPT1A in the liver. **(C)** Protein expression of CPT1A in the liver. **(D)** CPT1A activity in L-02 cells. **(E)** mRNA expression of CPT1A in L-02 cells. **(F)** Protein expression of CPT1A in L-02 cells. **(G)** Theoretical binding mode of API at the binding site of CPT1A. Data are expressed as means ± SEM (n = 6 in mice and n = 3 in L-02 cells); **p < 0.01, ***p < 0.001 compared to the control group; #*p* < 0.05, ##*p* < 0.01, ###*p* < 0.001 compared to the APAP group).

Furthermore, CPT1A activity, mRNA expression, and protein expression in L-02 cells were evaluated, and the results are presented in Figures 5D–F. The results demonstrated that API enhanced the activity of CPT1A *in vitro* in a dose-dependent manner. Therefore, we established that API could significantly increase the expression of CPT1A.

A computational protein/molecule docking analysis was performed to further investigate the relationship between API and CPT1A. A three-dimensional (3D) interaction map revealed that API interacted with the active conformation of CPT1A. API can interact with the active site of CPT1A with a binding energy of −7.6 kcal/mol to form four strong hydrogen bonds with amino acid residues including GLY 244, GLY 246, TYR 254, and TYR 241 of CPT1A (Figure 5G; Table1). API can bind efficiently to the activation site of CPT1A due to the ligand–protein interactions, which indicates that API could activate CPT1A activity.

**TABLE 1:** Docking score of apigenin with CPT1A and AMPK.

Protein	Docking score		
	Affinity (kcal/mol)	H-bond	Amino acid
CPT1A	−7.6	4	GLY 244
			GLY 246
			TYR 254
			TYR 241
AMPK	−9.0	3	LYS 45
			VAL 96
			VAL 96

CPT1A, carnitine palmitoyltransferase I; AMPK, AMP-activated protein kinase.

### Apigenin Activated AMP-Activated Protein Kinase Pathway Both *In Vitro* and *In Vivo*

The results have demonstrated that API can directly bind to CPT1A and subsequently promote its activity. Previous studies have demonstrated that AMP-activated protein kinase (AMPK) can activate CPT1A to protect the liver. Li et al. (2019) established that the AMPK pathway was the upstream regulator of CPT1A, and it could regulate CPT1A and fatty acid β-oxidation (FAO) (Li et al., 2019). In addition, a study by Tobita et al. (2018) revealed that an increase in the mRNA expression level of hepatic CPT1A was associated with Thr172 phosphorylation of AMPK α (AMPKα) in the liver, which alleviated hepatic steatosis (Tobita et al., 2018). Therefore, the AMPK pathway was investigated to further elucidate the role of AMPK in API treatment of APAP-induced hepatotoxicity.

Western blot results revealed that API can activate the AMPK pathway in mice liver and activate phosphorylation of AMPK and GSK, which is also part of the pathway. Further experiments on L-02 cells demonstrated that API activated p-AMPK in a dose-dependent manner, which suggested that API activated the AMPK pathway both *in vitro* and *in vivo.*

**FIGURE 6:**
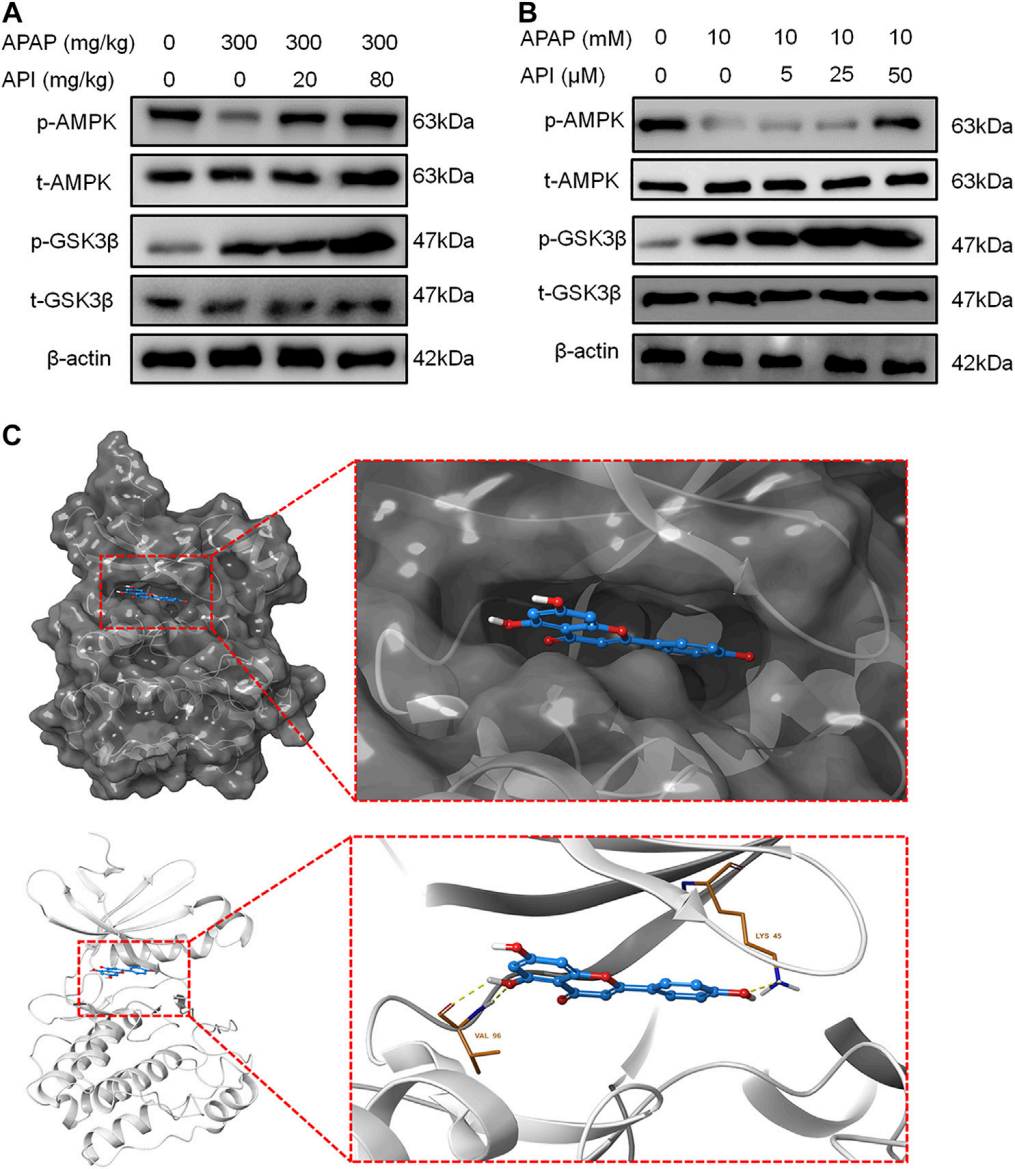
Apigenin (API) activated the AMP-activated protein kinase (AMPK) pathway both *in vitro* and *in vivo*. **(A)** Protein expression of AMPK and GSK-3β in mice liver. **(B)** Protein expression of AMPK and GSK-3β in L-02 cells. **(C)** Theoretical binding mode of API at the binding site of AMPK. Data are expressed as means ± SEM (n = 6 in mice and n = 3 in L-02 cells).

Molecular docking analyses further demonstrated that API interacted with the active site of AMPK with a binding energy of −9.0 kcal/mol (Table 1; Figure 6C). API formed hydrogen bonds with the amino acids LYS 45 and VAL96 of AMPK. The results revealed that API exhibited distinct affinities to the binding sites as an agonist of AMPK, which was consistent with *in vivo* and *in vitro* activities.

### Inhibition of AMP-Activated Protein Kinase by Compound C Reversed the Therapeutic Effect of Apigenin Both *In Vitro* and *In Vivo*

**FIGURE 7:**
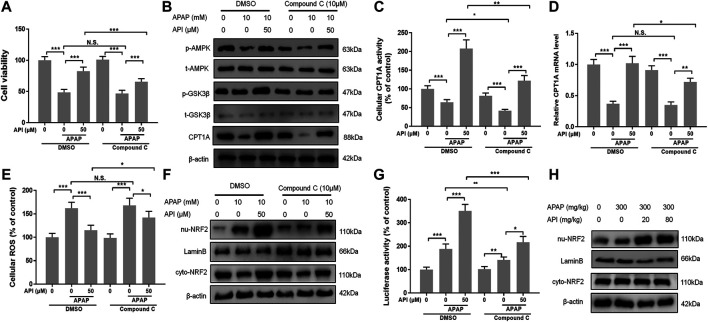
Inhibition of AMP-activated protein kinase (AMPK) by compound C reversed the therapeutic effect of API *in vitro*. **(A)** Cell viability evaluated by MTT assay after treatment with APAP (10 mM) and API or compound C in L-02 cells. **(B)** Protein expression of AMPK and GSK-3β in L-02 cells. **(C)** Carnitine palmitoyltransferase I (CPT1A) activity in L-02 cells. **(D)** mRNA expression of CPT1A in L-02 cells. **(E)** Cell reactive oxygen species levels. **(F)** Protein expression of cytosolic and nuclear factor erythroid 2–related factor 2 (NRF2) in L-02 cells. **(G)** NRF2 luciferase activity. **(H)** Protein expression of cytosolic and nuclear NRF2 in mice liver. Data are expressed as means ± SEM (n = 6 in mice and n = 3 in L-02 cells); *p < 0.05, **p < 0.01, ***p < 0.001.

Compound C, an inhibitor of AMPK (Vucicevic et al., 2011), was used to perform further experiments to verify if the therapeutic effect of API was activated through the AMPK pathway. We evaluated cell viability of L-02 cells treated with API or compound C using MTT assay, and the results demonstrated that compound C decreased the protection effect of API, while compound C in isolation had no effect (Figure 7A). Western blot results also revealed that compound C significantly decreased protein expression of p-AMPK and CPT1A (Figure 7B). A similar phenomenon was observed in CPT1A activity in cells and relative CPT1A mRNA levels (Figures 7C,D). Results of ROS generation in cells revealed that compound C inhibited antioxidative stress effect of API (Figure 7E).

Nuclear factor erythroid 2–related factor 2 (NRF2) is a transcription factor that regulates cellular defense by inducing the expression of various detoxiﬁcation and antioxidant genes (Ma, 2013), and AMPK exerts a positive effect on NRF2/heme oxygenase-1 (Zimmermann et al., 2015). In the present study, the AMPK downstream oxidative stress–related protein, NRF2, was analyzed by Western blot. The results revealed that API activated translocation of NRF2 into the nucleus, whereas compound C inhibited the promotion of NRF2 by activating API (Figure 7F). Furthermore, luciferase activity was evaluated, and results revealed that compound C significantly decreased API-induced NRF2 activity in L-02 cells (Figure 7G). Finally, the expression of NRF2 in mice liver tissues was verified by Western blot (Figure 7H), and it was established that API increased the expression of NRF2.

**FIGURE 8:**
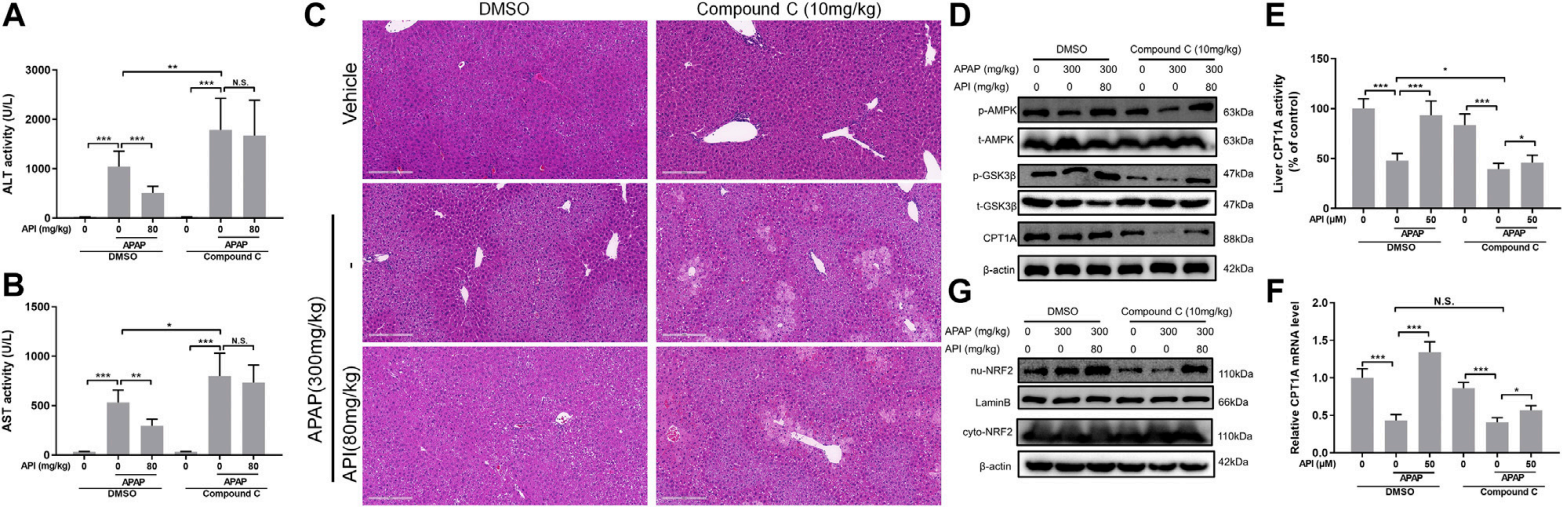
Inhibition of AMP-activated protein kinase (AMPK) by compound C reversed the therapeutic effect of apigenin (API) *in vivo*. **(A)** and **(B)** Evaluation of alanine aminotransferases and aspartate aminotransferases activities, respectively. **(C)** Representative images of H&E-stained liver sections (magnification of ×200). **(D)** Protein expression of AMPK, GSK-3β, and CPT1A in mice liver. **(E)** CPT1A activity in the liver. **(F)** mRNA expression of CPT1A in the liver. **(G)** Protein expression of cytosolic and nuclear factor erythroid 2–related factor 2 in mice liver. Data are expressed as means ± SEM (n = 6); *p < 0.05, **p < 0.01, ***p < 0.001.

Subsequently, the effect of compound C in APAP-induced mice liver injury was investigated. The results revealed that compound C aggregated hepatotoxicity induced by APAP and invalidated the effect of API (Figures 8A–C). Moreover, Western blot results were consistent with the *in vitro* data. Compound C decreased the phosphorylation level of AMPK and GSK-3β, as well as inhibited the levels of CPT1A and nuclear translocation of NRF2 (Figures 8D–8G).

## Discussion

DILI is the primary cause of ALF among European and American countries (Bhushan and Apte, 2019), and APAP is the principal cause of drug-induced dose-dependent liver injury. APAP has no toxic effects when used in appropriate doses. However, when an overdose of APAP is taken, its metabolite NAPQI accumulates and subsequently leads to depletion of GSH, which can form a complex with NAPQI to detoxify APAP. Consequently, intracellular mitochondrial and DNA damage occurs, and oxidative stress generation increases, eventually leading to liver necrosis (Mitchell et al., 1973; Kanno et al., 2017).

A previous study by Yang et al. established that an oral administration of API in mice for 7 days before administration of APAP did not offer protection against APAP-induced acute liver injury (Yang et al., 2013); however, clinical DILI is often acute. Therefore, seeking timely treatment, rather than a prophylactic one, is essential. The present study was aimed at investigating the amelioration function of API in APAP-induced hepatotoxicity both *in vitro* and *in vivo* and to investigate the potential mechanisms. Notably, in the animal experiments, API was administered 4 h after administration of APAP (300 mg/kg). During the period, APAP could have been completely metabolized to form NAPQI in mice (Corcoran et al., 1985), which demonstrated that the therapeutic mechanism of API was not induced by the inhibition of the conversion of APAP to NAPQI.

### Metabolomic Analyses of Cells and Serum Revealed an Aberrant Level of Carnitine Palmitoyltransferase I

The primary route for lipid expenditure is through mitochondrial FAO, which is an essential process in which free fatty acids are esterified with CoA, transported into the mitochondrial matrix, and oxidized to generate acetyl-CoAs (van der Windt et al., 2012). CPT1A is one of the proteins in the CPT system, which is responsible for converting acyl-CoAs into acylcarnitines, and is transported across the mitochondrial membranes by translocase and converted back to acyl-CoAs by CPT2 inside the mitochondria before β-oxidation occurs (Bonnefont et al., 2004). Previous studies have revealed that an increase in CPT1 activity promoted fatty acid oxidation and decreased steatosis (Derdak et al., 2013). The present study has revealed that not only API can promote CPT1A activity and exert an anti-liver injury effect but also it can also interact with the active site of CPT1A. Evaluation of CPT1A activity in isolated mitochondrial extracts could be performed to further validate the results of the present study because CPT1 is anchored on the outer mitochondrial membrane (Dai et al., 2018).

AMPK is a central hub that controls the energy metabolism by interacting with AMP and phosphorylation *via* an upstream kinase (Konrad et al., 2005). AMPK has anti-inflammatory eﬀects, regulates catabolism and anabolism, and improves the redox balance (Hayashi et al., 2000; Levine et al., 2007). The AMPK pathway is the upstream regulator of CPT1A, and it can regulate CPT1A and fatty acid β-cooxidation (Li et al., 2019). The current study demonstrated that API enhanced activation of the AMPK pathway and reversed APAP-induced hepatotoxicity. The effect could be reversed by compound C, an AMPK inhibitor. However, only *in vitro* experiments were performed; therefore, further studies should be conducted using *in vivo* experiments.

Furthermore, the AMPK pathway regulates several other cellular processes, including lipid and glucose metabolism and autophagy (Rabinovitch et al., 2017; Herzig and Shaw, 2018). API could enhance the protective effect of AMPK in APAP-induced hepatotoxicity, and previous studies have demonstrated that autophagy plays a crucial role in the repair of damaged hepatocytes (Ding et al., 2010; Komatsu et al., 2010). We therefore hypothesized that API can activate the process of autophagy that is associated with AMPK, a hypothesis that requires further study in the future. In conclusion, the study has demonstrated that API protects against APAP-induced hepatotoxicity by activating the AMPK/CPT1 pathway.

## Materials and Methods

### Reagents and Chemicals

API (purity >98.5%) was purchased from Meilune (Dalian, China). Kits for ROS, MDA, and MPO were purchased from Nanjing Jiancheng Bioengineering Institute (Nanjing, China). Lipofectamine RNAiMAX, 2′-7′-dichlorodihydrofluorescein diacetate (H_2_DCFDA), RPMI1640, and fetal bovine serum (FBS) were purchased from Life Technology (Carlsbad, CA, United States). NE-PER nuclear, cytoplasmic extraction reagents, and a Pierce BCA Protein Assay Kit were purchased from Thermo Fisher Scientific (Waltham, MA, United States). A whole-cell protein extraction and enhanced chemiluminescence kits were obtained from Millipore (Darmstadt, Germany). PrimeScript RT Master Mix and SYBR Premix Ex Taq were purchased from TaKaRa (Shiga, Japan). Antibodies for immunoblotting, including anti-actin, -CPT1A, -Phospho-AMPKα1, -AMPKα1, -Phospho-GSK-3β, -GSK-3β, -NRF2, and -LaminB were purchased from Cell Signaling Technology (Danvers, MA, United States). Methanol and acetonitrile (HPLC grade) were purchased from Thermo Fisher Chemicals (Waltham, MA, United States). APAP, NAPQI, and compound C were purchased from Sigma-Aldrich (St. Louis, MO, United States). Fenclonine was purchased from Aladdin (Shanghai, China).

### Animals and Treatments

C57BL/6J mice (20 g ± 2) were obtained from the Shanghai Laboratory Animal Center of the Chinese Academy of Sciences (Shanghai, China). Experimental animals were given water and fed on ad libitum diet. Animals were housed in rooms maintained at a constant temperature of 20–25°C and a humidity of 65 ± 5% with a 12-h light–dark cycle. All animals received humane care in compliance with the institutional animal care guidelines approved by the Experimental Animal Ethical Committee of Shanghai University of Traditional Chinese Medicine. The protocol was reviewed and approved by the Experimental Animal Ethical Committee of Shanghai University of Traditional Chinese Medicine (Permit Number: PZSHUTCM200327006). All surgeries were performed under sodium pentobarbital anesthesia, and efforts were made to minimize suffering.

Twenty-four mice were divided randomly into four groups: 1) control group (vehicle), 2) model group (APAP = 300 mg/kg), 3) treatment group (APAP = 300 mg/kg + API = 20 mg/kg), and 4) APAP (300 mg/kg) + API (80 mg/kg) group. APAP or saline (vehicle) was orally administered to the mice after depriving them of food and water for 12 h, which was followed by administration of API (20 or 80 mg/kg) after 4 h. Mice were euthanized 8 h after APAP administration (4 h after API administration), and blood and liver samples collected.

To evaluate the role of time course in APAP-induced hepatotoxicity, mice were divided randomly into three groups: Twenty-four mice were divided randomly into four groups: 1) control group (vehicle), 2) model group (APAP = 300 mg/kg), and 3) treatment group (APAP = 300 mg/kg + API = 80 mg/kg). APAP or saline (vehicle) was orally administered to the mice after depriving them of food and water for 12 h, which was followed by administration of API (80 mg/kg) after 4 h. Mice were euthanized 4 and 16 h after APAP administration (0 and 12 h after API administration), and blood and liver samples were collected.

To evaluate the role of AMPK in regulating APAP-induced hepatotoxicity, mice were divided randomly into six groups: 1) DMSO, 2) DMSO + APAP (300 mg/kg), 3) DMSO + APAP (300 mg/kg) + API (80 mg/kg), 4) compound C (10 mg/kg), 5) compound C (10 mg/kg) + APAP (300 mg/kg), and 6) compound C (10 mg/kg) + APAP (300 mg/kg) + API (80 mg/kg). Mice were injected intraperitoneally with compound C after depriving them of food and water for 12 h, which was followed by administration of APAP or saline 1 h after administration of compound C. Afterward, API was administered 5 h after treatment with compound C. Mice were euthanized 9 h after compound C administration, and blood and liver samples were collected.

### Cell Culture and Viability Assay

The L-02 cell lines were purchased from the Shanghai Institute of Cell Biology (Shanghai, China) and cultured in DMEM with FBS and 1% antibiotics. Cells were placed into 96-well plates at an initial density of 5,000 cells per well. After attachment, cells were pretreated with API or compound C (10 *μ*M) for 15 min and finally incubated with APAP or NAPQI for 48 h. After treating cells with 500 μg/ml MTT for 4 h, the resulting formazan blue was dissolved in 10% SDS, 5% isobutanol, and 0.01 M HCl, and plates were scanned using a microplate reader (Thermo Fisher Scientific, Waltham, MA, United States) at a wavelength of 570 nm, with 630 nm as a reference wavelength. Cell viability was standardized as a percentage of the control.

### Measurement of Reactive Oxygen Species in the Liver and Cells

ROS in cells was measured using an H2DCFDA probe as described previously (Zhao et al., 2019). To measure ROS levels in the liver, cold liver homogenate was centrifuged (10,000 g, 15 min, 4 °C); supernatants were incubated with 10 μM H_2_DCFDA in the dark for 1 h and subsequently transferred to a black-walled clear-bottomed 96-well plate. Fluorescence was immediately read at an excitation wavelength of 485–720 nm and an emission wavelength of 525–720 nm using a Synergy H4 spectrophotometer (BioTek, Winooski, VT, United States). Protein concentrations of the supernatants were quantified using BCA protein assay kits, calculated as units of fluorescence per microgram of protein, and concentrations presented as percentages of controls (% control).

### Molecular Docking Analysis

Molecular docking analyses were performed to investigate interactions between API, AMPK, and CPT1A using AutoDock Vina 1.1.2 (Trott and Olson, 2010). A 3D structure of API was drawn using ChemBioDraw Ultra 14.0 and converted to a 3D structure by ChemBio 3D Ultra 14.0. The 3D coordinates of AMPK (PDB ID: 5EZV) were retrieved from the RCSB Protein Data Bank. The homology model was obtained from SWISS-MODEL (https://www.swissmodel.expasy.org/), and ligand binding sites of the proteins were predicted by POCASA 1.1 in which the principle was consistent with AutoDock software (Biasini et al., 2014) due to the lack of CPT1A protein structure in the PDB database. AutoDockTools version 1.5.6 (Sanner, 1999; Trott and Olson, 2010) was employed to generate docking input files. The crystallographic ligands were extracted and fed into a docking database for redocking, and hydrogen atoms added. An auxiliary program AutoGrid was used to generate a docking area that was defined as a 40 × 40 × 40 3D grid centered on the ligand binding site with a 0.375 Å grid space. All bond rotations for the ligands were ignored in this study. The best scoring pose from Vina docking score evaluations was selected for further analyses using PyMoL 1.7.6 software.

### Statistical Analysis

Data were analyzed using IBM SPSS Statistics version 21.0 (IBM Corp., Armonk, NY, United States). One-way ANOVA with least significant difference *post hoc* tests was used to compare means between groups. Data were expressed as mean ± SEM. *p* < 0.05 was considered statistically significant. More detailed materials and methods are in the Supplementary Material.

## Data Availability Statement

The raw data supporting the conclusions of this article will be made available by the authors, without undue reservation.

## Ethics Statement

All animals received humane care in compliance with the institutional animal care guidelines approved by the Experimental Animal Ethical Committee of Shanghai University of Traditional Chinese Medicine

## Author Contributions

JZ, XL, JL, and HY performed the experiments. CH and LL designed the research study. JZ and FL collected the data. JZ and CH analyzed the data. JZ and XL wrote the manuscript.

## Funding

This work was financially supported by the Natural Science Foundation of Shanghai (19ZR1451800), Budget of Experiment Center for Science and Technology (18LK022), and Guoyiqiangyou Foundation of Shanghai Hongkou (No. HGY-KY-2018–03 and HGY-MGB-2018–01–05).

## Conflicts of Interest

The authors declare that the research was conducted in the absence of any commercial or financial relationships that could be construed as a potential conflict of interest.
