# Supplementary material for: Apigenin Attenuates Acetaminophen-Induced Hepatotoxicity by Activating AMP-Activated Protein Kinase/Carnitine Palmitoyltransferase I Pathway
Source: Front Pharmacol. 2020 Nov 20;11:549057. doi: 10.3389/fphar.2020.549057 (PMC7919381; doi:10.3389/fphar.2020.549057)
Supplement: Supplementary file 1 [file DataSheet1_v1.DOCX]

**Apigenin attenuates acetaminophen‑induced hepatotoxicity by activating AMPK/CPT1 pathway**

Jiaqi Zhang^a,1^, Xiaoqing Liang^c,1^, Jiacheng Li^a^, Hao Yin^a^, Fangchen Liu^d^, Cheng Hu^b, *^, Ling Li^a, *^

^a^ *Shanghai TCM-Integrated Institute of Vascular Anomalies, Shanghai TCM-Integrated Hospital, Shanghai University of Traditional Chinese Medicine, Shanghai, 200082, China.*

^b^ *Experiment Center for Science and Technology, Shanghai University of Traditional Chinese Medicine, Shanghai, 201203, China.*

^c^ *Longhua Hospital Affiliated to Shanghai University of Traditional Chinese Medicine, Shanghai 200032, China.*

^d^ *Department of Neurology, Shanghai TCM-Integrated Hospital, Shanghai University of Traditional Chinese Medicine, Shanghai, 200082, China.*

** Corresponding authors. Tel.: +86 13601838793.*

*E-mail addresses:*

*Ling Li: lingli_z163@163.com.*

*Cheng Hu: hucheng10200@163.com.*

^1^ These authors contributed equally to this work.

**Materials and Methods**

**Collection of blood and tissue samples and biochemical analyses**

Liver tissue samples were fixed in 10% neutral buffered formalin and paraffin sections prepared. Liver sections were stained with hematoxylin–eosin (H&E) stain to assess liver damage under a microscope. Blood samples were kept at room temperature for 2 h and subsequently centrifuged at 3000 rpm for 10 min to collect serum. ALT and AST activities were quantified, and liver MDA and MPO examined using commercial kits in accordance with standard protocols.

**Histological analysis of liver tissues**

Mice liver tissue samples were fixed in 10% phosphate buffered saline (PBS)-formalin for at least 24 h and embedded in paraffin for histological analyses. Samples were sectioned (5 μm), stained with H&E or Sirius Red stain according to standard protocols. Liver sections were then examined microscopically for structural changes and observed under a light microscope (Olympus, Tokyo, Japan) to evaluate liver damage.

**Protein extraction and western blot analysis**

A-50 μg sample of protein from lysed cells was separated using 10% SDS-PAGE, transferred to a nitrocellulose membrane, and blocked for 2 h. Membranes were incubated overnight with primary antibodies, followed by horseradish peroxidase (HRP)-conjugated secondary antibodies, and protein bands were visualized using ECL Plus detection reagent.

**RNA isolation and quantitative real-time PCR**

RNA was isolated from PTC cells using TRIzol reagent, and cDNA was synthesized from 1 μg of total RNA in a 21 μl reaction volume using oligo (dT) 18 primers and SuperScript reverse transcriptase. PCR amplification was carried out using Taq DNA polymerase with 1 μl of first-strand cDNA as templates. Thermal cycling was performed for 30 s at 94°C, 30 s at 55°C, and 30 s at 72°C for 30 cycles, and relative expression levels were calculated using the 2^˗ΔΔCT^ method.

**Metabolomics analysis**

**Extraction and derivation of cell samples**

Logarithmic growth phase cells were inoculated into 6-well plates at a density of 5×10^5^ cells per well and treated according to the method previously described. The following steps were performed: (1) after 24 h of culturing, the supernatant was discarded and cells placed on ice; (2) cells were washed with 4 mL PBS, 2 mL of cold methanol was added to terminate metabolism, and cells were collected by scraping; (3) cells were lysed using a cell pulveriser for metabolite extraction and samples placed on ice for 20 min; (4) supernatants were transferred to an Eppendorf (EP) tube after centrifugation at 14,000 g for 10 min, which was followed by addition of 10 μL DL-o-chlorophenylalanine (2.9 g/L, internal standard). The supernatant was dried with nitrogen and the residue stored at -80°C; (5) the residue was reconstituted with 100 μL of methanol before analysis.

**Preparation of serum samples**

Serum samples (100 μL) were deproteinised using 400 μL of methanol containing internal standards (29 μg/mL DL-o-chlorophenylalanine). Samples were vortexed for 30 s and centrifuged at 12,000 rpm at 4°C for 15 min. A-200 μL sample of supernatant was transferred to a vial for analysis.

**UPLC-Q-Orbitrap-MS analysis**

Liquid chromatographic separation was achieved at a flow rate of 0.3 mL/min on an high strength silica (HSS) T3 column (100 mm × 2.1 mm, 1.8 μm) using 0.1% formic acid (buffer A) and acetonitrile (buffer B). Gradient elution was performed as follows: 0-2 min, 95% A; 2°12 min, 5% A; 12°15 min, 5% A; 15°17 min, 95% A. MS data acquisition was achieved using both positive and negative ionisation modes. Compounds were detected by full-scan mass analyses from 50 to 1000 m/z at a resolution of 60,000 FWHM. Electrospray ionisation (ESI) parameters were as follows: positive mode, (1) heater temperature = 300°C, (2) sheath gas flow = 45 arb (arbitrary units), (3) auxiliary gas flow = 10 arb, (4) sweep gas flow = 0 arb, (5) electrospray voltage = 3.8 kV, (6) capillary temperature = 350°C, (7) S-LensF level = 30%; negative mode, (1) heater temperature = 300°C, (2) sheath gas flow = 45 arb, (3) auxiliary gas flow = 5 arb, (4) sweep gas flow = 1 arb, (5) electrospray voltage = 3.2 kV, (6) capillary temperature = 350°C, and (7) S-LensF level = 60%.

**Metabolic data analysis**

LC-MS data were extracted and pre-processed by Compound Discoverer 2.1 software (Thermo Fisher Scientific, Waltham, MA, USA), and exported to generate a matrix including mass, retention time and peak intensity. Multivariate statistical analyses, including principal component analysis (PCA) and orthogonal partial least squares discriminant analysis (OPLS-DA) were performed on both serum and cell samples of mice in each group using SMICA-P software.

**Metabolite identification and pathway analysis**

Metabolites were identified based on accurate masses and product ion spectra by searching the HMDB, KEGG and mzCloud databases. Metabolomic pathway analyses were performed using MetaboAnalyst 4.0.

**Carnitine palmitoyltransferase 1A (CPT1A) activity assay**

The activity of CPT1A was evaluated using a spectrophotometer. Liver samples were homogenized in a buffer (0.25 sucrose, 1 mM EDTA, 0.1% ethanol) supplemented with Halt Protease Inhibitor Cocktail (Thermo Fisher Scientific, Rockford, IL, USA), followed by centrifugation at 300 x g at 4°C for 10 min. Afterwards, the supernatant was further purified by a second centrifugation at 12,000 x g at 4°C for 15 min. A-50 μl sample of the precleared supernatant was mixed with 50 μl of Tris-5,5’-dithio-bis-(2-nitrobenzoic acid) (DTNB) buffer (116 mM Tris, 2.5 mM EDTA, 2 mM DTNB, 0.2 % Triton X-100, pH 8.0) in a 96-well plate. The mixture was preincubated for 5 min at room temperature and 50 μl of 1 mM palmitoyl-CoA added to the well. The reaction was initiated by adding 5 μl of L-carnitine solution (1.2 mM, dissolved in 1 M Tris, pH 8.0), and release of CoA-SH from palmitoyl-CoA was monitored using a spectrophotometer at a wavelength of 412 nm for 180 s using a SpectraMax M5 multi-mode microplate reader (Molecular Devices, Sunnyvale, CA, USA). A CoA-SH (Sigma-Aldrich, St. Luis, MO, USA) standard curve (1-200 nmol/well) was generated and the activity of CPT1 presented as a percentage of the controls (% control).

**NRF2 luciferase reporter assay**

NRF2 luciferase reporter assay was performed according to the manufacturer’s instructions. L-02 cells were transfected with NRF2 transcription response element (TRE) containing a construct using lipofectamine 3000. After 24 h of transfection, cells were pre-treated with baicalein or baicalin for 15 min, and subsequently incubated with APAP for another 24 h. Luciferase activities were quantified using the Duan-Glo Luciferase Assay System, and the constitutively expressed Renilla luciferase was used as an internal control.
