# Supplementary material for: Apigenin Attenuates Acetaminophen-Induced Hepatotoxicity by Activating AMP-Activated Protein Kinase/Carnitine Palmitoyltransferase I Pathway
Source: Front Pharmacol. 2020 Nov 20;11:549057. doi: 10.3389/fphar.2020.549057 (PMC7919381; doi:10.3389/fphar.2020.549057)
Supplement: Supplementary file 2 [file DataSheet2_v1.DOCX]

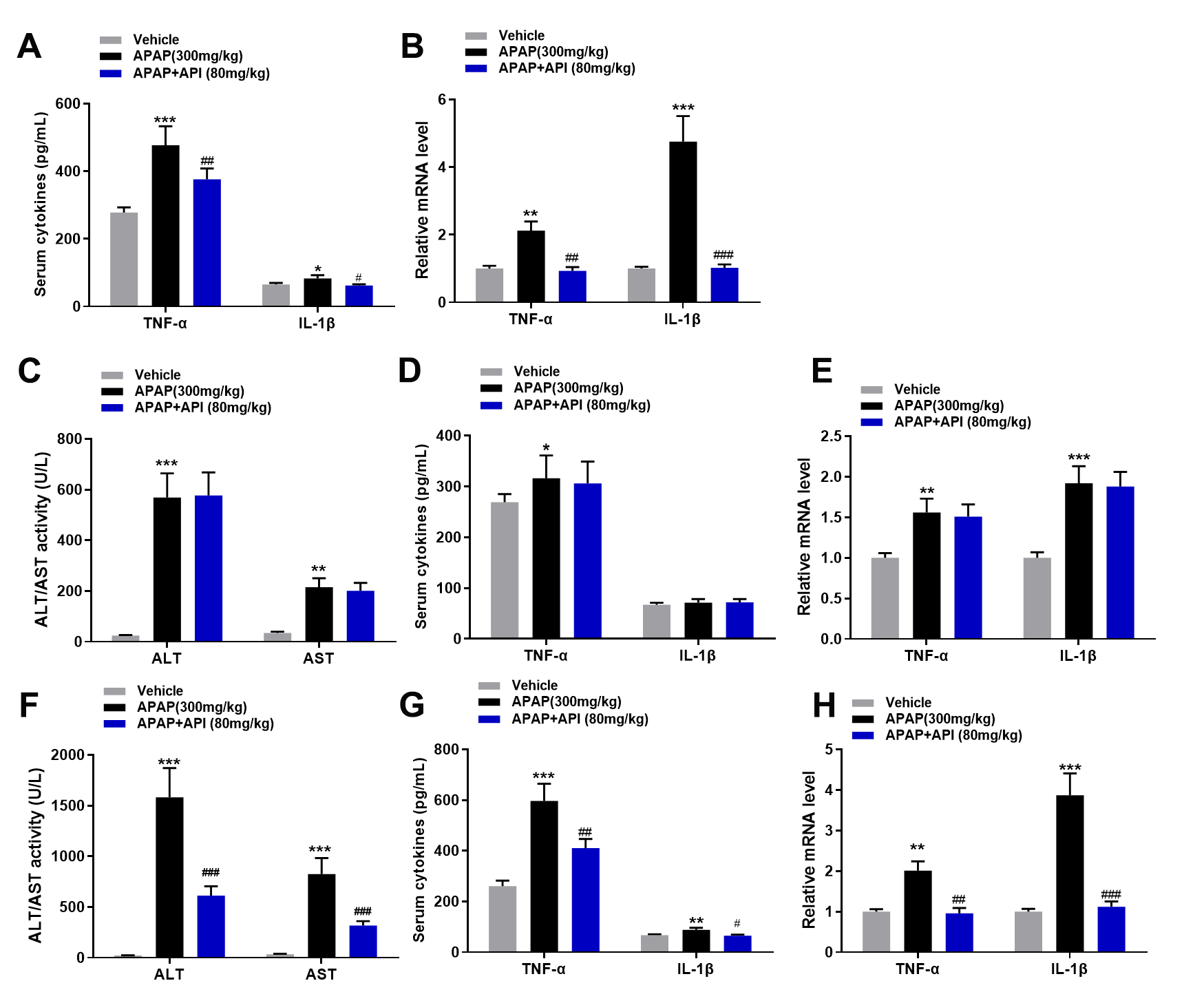


Figure Legend

Supplementary Fig.1 A. IL-6 and TNF-α concentration in mice serum 4 h after API administration. B. IL-6 and TNF-α mRNA level in mice liver tissues 4 h after API administration. C. ALT and AST activities in mice serum 0 h after API administration. D. IL-6 and TNF-α concentration in mice serum 0 h after API administration. E. IL-6 and TNF-α mRNA level in mice liver tissues 0 h after API administration. C. ALT and AST activities in mice serum 12 h after API administration. D. IL-6 and TNF-α concentration in mice serum 12 h after API administration. E. IL-6 and TNF-α mRNA level in mice liver tissues 12 h after API administration. Data are expressed as means ± SEM (n = 6 in mice); *p <0.05,**p <0.01, ***p <0.001 compared to control group; #p <0.05, ##p <0.01, ###p <0.001 compared to APAP group).
